# Digital economy and pollution reduction–Mechanism and regional heterogeneity

**DOI:** 10.1371/journal.pone.0277852

**Published:** 2023-02-10

**Authors:** Zhibo Yang, Liyou Fu, Yirong Chen

**Affiliations:** Business School, Shanghai Dianji University, Shanghai, China; Zhejiang University of Finance & Economics, CHINA

## Abstract

The digital economy and ecological environment are two major issues related to high-quality economic development. Scholars have not yet reached a unified conclusion about the link between the digital economy and pollution emissions, and the impact mechanism of the former on the latter needs further study. Using data from 278 Chinese cities from 2010 to 2019, this research employs coupling coordination analysis, fixed effect analysis and mediation analysis to examine the heterogeneous impact mechanisms of the expansion of the digital economy on urban pollution reduction from many angles. It discovers that, first, the growth of the digital economy has decreased the discharge of urban pollutants overall. Second, the impact mechanisms of the digital economy are heterogeneous. From a regional perspective, industrial structure supererogation plays an intermediary role in the relationship between digital economy development and pollution reduction in the eastern and central regions, but the mediating effect is not significant in the western and northeastern regions. In terms of the city development level, industrial structure supererogation has significantly mediated the relationship between the growth of the digital economy and the reduction of pollution in first- and second-tier cities, but this mediating effect is not significant in third-tier and other cities. Third, the above conclusions are still valid after the robustness test is carried out using instrumental variable estimation, replacement of the estimation method, and replacement of explanatory variables. This study is a useful contribution to research on the effects of the digital economy and the factors influencing pollution reduction. The results advance the study of the digital economy and also have practical implications for improving China’s ecological environment and fostering high-quality economic growth. Finally, we provide policy suggestions for the coordinated promotion of the digital economy’s development, industrial structure supererogation and environmental pollution reduction.

## Introduction

Environmental pollution is the greatest challenge facing the world in the 21st century. The ecological environment serves as the foundation for both human survival and nations’ sustainable growth. Although China’s economy has grown quickly over the past 40 years, the old high-input, high-consumption, high-emission growth model has also experienced significant ecological and environmental challenges, making the development of an ecological society a clear priority. Problems such as dwindling resources, environmental constraints and ecosystem degradation are increasingly prominent. China’s economic growth mode is currently transitioning from high-speed growth to high-quality development, and accelerating green development has become imperative. Since the 18th National Congress of the CPC(Communist Party of China), China has successively issued dozens of reform plans pertaining to the development of ecological civilization. The main principles, key tasks, institutional guarantees and other aspects have been comprehensively specified, and a sensible plan for creating a beautiful China has been drawn up. In the digital economy, data resources are the primary element of production, a new generation of information network is the primary medium, digital transformation of all factors is a significant driving force, and digital technology application is the main feature. It is a crucial instrument for the world to achieve sustainable development. As a new economic form, it continues to penetrate into all areas of society and the economy and has become a catalyst for the transformation and development of both and the main track for a new wave of worldwide industrial competition. Amidst the gradual penetration of information technology into all spheres of society, using the digital economy to promote green and sustainable economic growth has become an important theoretical and practical issue.

## Literature review

There are currently three main views on how the digital economy affects pollution emissions. First, it can reduce and inhibit emissions, thereby improving environmental quality [1–3]. Chatti and Muhammad found that digital technology adoption can reduce environmental pollution for both developed and developing countries, and telephone use is more efficient than mobile phones and internet technologies in urban areas [4]. Latif et al. argued that investment in the ICT industry decreases the likelihood of air pollution and increases environmental quality [5]. The second view is that digital technology can increase the emission of pollutants because the use of modern ICT such as AI, CPS, and IOTs has increased energy consumption; hence, the rise of the digital economy can increase pollution emissions [6–8]. Lennerfors et al. noted that as ICT becomes more prevalent, ICT equipment will consume a large amount of energy every year and pose a greater threat to the environment [9]. Cheng et al. contended that information technology has significantly aggravated environmental pollution because of the rebound effect [10]. The third point of view is that there is a nonlinear relationship between information technology adoption and pollution. Some scholars have pointed out that the emission of pollutants shows an increasing trend at the beginning of digital economy development but decreases in the later stage [11, 12]. The above research demonstrates that researchers have not come to a consensus regarding the connection between digital economy growth and environmental pollution. This relationship is affected by factors such as the stage of economic development, industry characteristics, and regional resource endowment [4, 13]. Additional research is needed to reveal how the development of the digital economy will affect the reduction of environmental pollution.

In addition, existing research shows that the digital economy sector offers a substantial impetus to optimize and modernize the industrial structure [3, 13–16]. First, it provides technical support to traditional industries and improves old production and organization methods; it also makes traditional industries more efficient and enhances the technical content of their products, leading to the upgrading of traditional industries [17]. Second, as new business models and sectors emerge more rapidly as a result of the convergence of traditional and digital economy sectors, the industrial system will become more advanced [18, 19]. Finally, the growth of the digital economy sector has altered the market’s demand structure and the industrial structure. The relationship between industrial structure supererogation and pollution reduction is ambiguous. Some scholars have pointed out that industrial structure supererogation is an effective way to prevent and reduce pollution at its source. It can reduce the proportion of heavy industry while also promoting consumption upgrades and reducing environmental pollution [20]. However, Wang et al. highlighted that the inhibition effect of industrial structure supererogation on water pollution shows a law of first increasing and then decreasing. Other research has also found that industrial structure supererogation and environmental pollution are negatively or nonlinearly related [21]. With regard to the relationship of digital technology, industrial structure supererogation and environmental pollution, Chen et al. pointed out that technological innovation can improve industrial energy efficiency and significantly reduce pollution emissions [22]. Huang et al. claimed that technology innovation has a strong positive effect on industrial structure upgrading, and industrial structuring has always demonstrated a positive effect on ecological efficiency [23]. However, Danish et al. asserted that ICT can boost industrial development, ultimately increasing energy consumption, which exacerbates environmental pollution [24]. In summary, while some studies have explored the effects of the digital economy on the environment, most previous studies, which are practically theoretical research, have used specific digital technology applications, such as IOTs, to analyse the reduction effects of pollution emissions, and very few have examined the effects of the development of the digital economy on environmental pollution from both theoretical and empirical perspectives [3, 6]. There is no consensus on these effects, and the impact mechanism is not clear. Therefore, many urgent questions remain to be answered for China: What is the current coupling and coordination relationship among China’s digital economy development, industrial structure supererogation and pollutant emission reduction? Can the growth of the digital economy lower pollutant emissions? If so, what are the pathways and mechanisms by which this effect occurs? Does industrial structure supererogation have a mediating role between the digital economy and emission reduction? Is there any heterogeneity in the relationship among China’s digital economy development, industrial structure supererogation and pollutant emission reduction? This paper aims to further analyse the impact mechanism of the digital economy on environmental pollution reduction and the mediating role of industrial structure supererogation in China. The discussion of these issues advances the study of the digital economy and has practical implications for improving China’s ecological environment and fostering high-quality economic growth.

**Fig 1:**
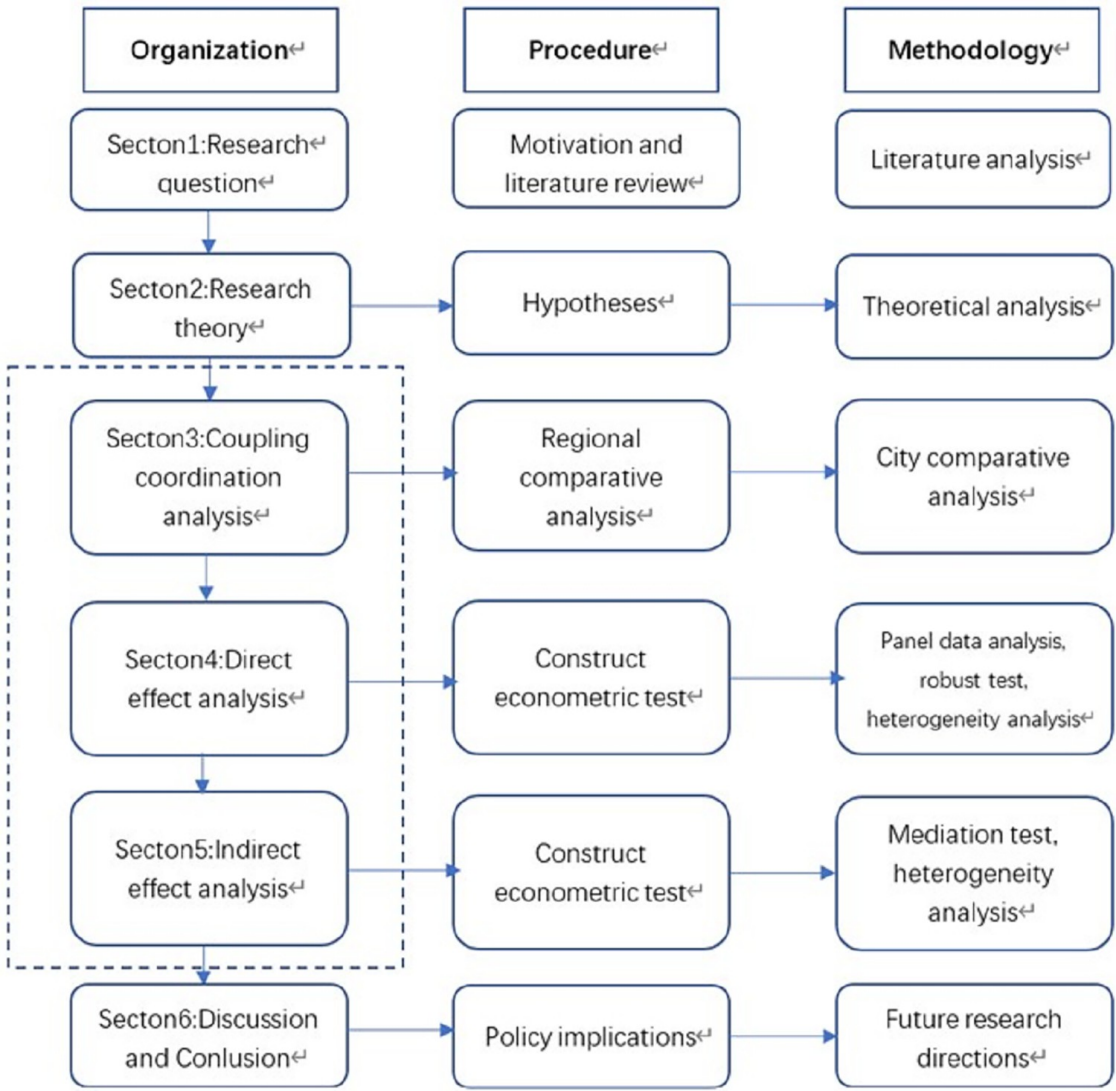
Logic of this research.

This research is organized as follows. The research topics and background work are provided in the first section. The research hypotheses are presented in the second section based on theoretical analysis. The third section analyses the degree of coupling and coordination among the digital economy, industrial structure supererogation and pollution emission reduction in different regions and cities of different development levels in China and constructs a digital economy and pollution emission reduction index using the entropy weight analysis method. The fourth section contains the analysis of the direct effect and its heterogeneity using the panel data analysis method. The fifth section analyses the mediating role played by industrial structure supererogation and the heterogeneity of this role. The sixth section presents the discussion, summary and policy suggestions. Fig 1 depicts the textual reasoning for this investigation.

## Theoretical analysis and hypothesis development

A comprehensive literature review was conducted using publications related to the topics of the digital economy, industrial structure supererogation, and pollutant emission reduction. Combining previous research on direct and indirect effects, this study analyses how the digital economy influences pollution emission reduction and proposes corresponding hypotheses.

### Direct effect analysis

In recent years, digital technologies represented by big data, AI, 5G, and IOTs have gradually been integrated into the field of energy saving and environmental protection. The growth of the digital economy has been crucial in fostering the promotion of energy conservation, reducing emissions, and even establishing a green economic system. Its influence on environmental pollution reduction has mainly been promoting the upgrading of the industrial structure, helping companies build green production models, reducing energy input intensity by enhancing energy efficiency, and increasing the efficiency of government regulation of environmental pollution [1–5]. First, the digital economy can reshape and restructure the traditional industrial ecological system. Guo et al. found that the expansion of the digital economy promotes the growth of a wide variety of emerging industries, such as big data, cloud computing, IoT, and AI, which are good for the establishment of new sectors [25]. The combination of new production factors such as big data, cyber-physical systems and new information infrastructure can accelerate the convergence of the digital economy and traditional sectors [13–15, 17]. Second, enterprises can reorganize their product and process data for analysis to improve productivity and reduce the use of high-polluting production factors, thereby reducing the emission of pollutants such as sulphur dioxide and soot in industrial production [17–19]. Third, with regard to energy use, the digital economy has the potential to construct a new power system with informatization and digitization. While doing so, relying on IOTs and AI to enhance the digital transmission capabilities of the power grid will speed up energy industry transformation and upgrading. Continuously encouraging the use of renewable energy, accelerating the replacement of traditional fossil energy consumption, and enabling the optimization and upgrading of the energy production and consumption structure will result in significant pollution emission reduction [26, 27]. Finally, digital technology can help accurately identify and track emerging pollution discharge problems in a timely manner. Big data, satellite remote sensing, IOTs and other technologies make it possible to more accurately identify and locate key areas such as densely populated urban areas, high energy consumption-bearing areas, ecologically sensitive areas, and environmental pollution areas and to find and address problems in a timely manner [3, 28–30]. Although some studies have noted that the application of ICT may increase energy consumption, thereby increasing pollutant emissions [6–8], because China’s economy has always been dominated by industry, the positive impact of ICT application will exceed its negative impact on pollution emissions. Therefore, we propose the following hypothesis:

H1: The growth of the digital economy is good for reducing the emission of urban environmental pollutants.

### Indirect effect analysis

Studies have already demonstrated that the growth of the digital economy has a substantial effect on industrial structure supererogation and is a key strategy for reducing environmental pollution [22, 23]. Industrial structure supererogation is fundamentally the process by which comparatively advanced industries in the industrial system eventually become the dominant industries, with a high technology development trend. Through digital industrialization and industrial digitalization, the digital economy propels the supererogation of the industrial structure. On the one hand, after decades of information technology development, the software, electronic and information technology service industry has become relatively mature and an important part of the industrial system. In recent years, IOTs, big data, AI and other information-related sectors have developed rapidly and are leading the growth of the digital economy. As a new economy, the digital economy allocates resources based on the internet platform, giving birth to new industries. These new industries enrich the industrial structure by transforming traditional industries through industrial associations, technology diffusion and other effects, which provide a path for upgrading traditional industries to elevate the industrial structure [13, 14]. On the other hand, the digital economy may make use of IoT, AI and other information technologies to offer crucial technical support to upgrade and transform traditional manufacturing enterprises. Integrating digital technology into all aspects of the production line can greatly promote the effective use of resources and improve work efficiency and product accuracy, thereby improving product quality, transforming products from low value added to high value added, and facilitating the transformation of traditional industries’ internal structure [15]. The digital economy also helps all types of enterprises increase the space to create and increase value through forms such as product and business model innovation, both of which support the modernization and transformation of traditional industrial structures [3, 16].

Scholars have conducted many theoretical and empirical explorations of the relationship between industrial structure supererogation and pollution emission reduction. Most think that they are positively correlated; that is, industrial structure supererogation has an obvious emission reduction effect. For example, Qiu and Tan used the data of 247 Chinese cities from 2000 to 2012 to conduct an empirical analysis and found that optimizing and upgrading the industrial structure is conducive to energy savings and pollution reduction [31]. According to empirical research by Yu et al. using Chinese coastal data from 11 provinces from 2006 to 2015, industrial structure supererogation will not only considerably reduce local pollution emissions but also spill over to adjacent areas [32]. Thus, we propose the following hypothesis:

H2: Industrial structure supererogation has a mediating effect on the relationship between the development of the digital economy and pollution reduction.

However, other academics contend that industrial structure supererogation and environmental pollution are negatively or nonlinearly related. For instance, Llop analysed the changes in major pollutants caused by changes to the industrial structure in Spain from 1995 to 2000 and concluded that the structural changes increased the number of pollutants [33]. Dinda asserted that there is an inverse U-shaped link between industrial structure supererogation and pollution [34]. Using a spatial econometric model and Chinese provincial data from 2003 to 2018, Ma and Cao found that the reduction effect was not significant in the eastern region and even increased pollution in the central and western regions [35]. The above research shows that the influence of industrial structure supererogation on pollution reduction may be related to other factors, such as local resource endowment, local economic development level, and original local industrial structure, which are closely related to local features. Therefore, we make the following hypotheses:

H3: There is regional and city heterogeneity in the mediating effect of industrial structure supererogation.

**Fig 2:**
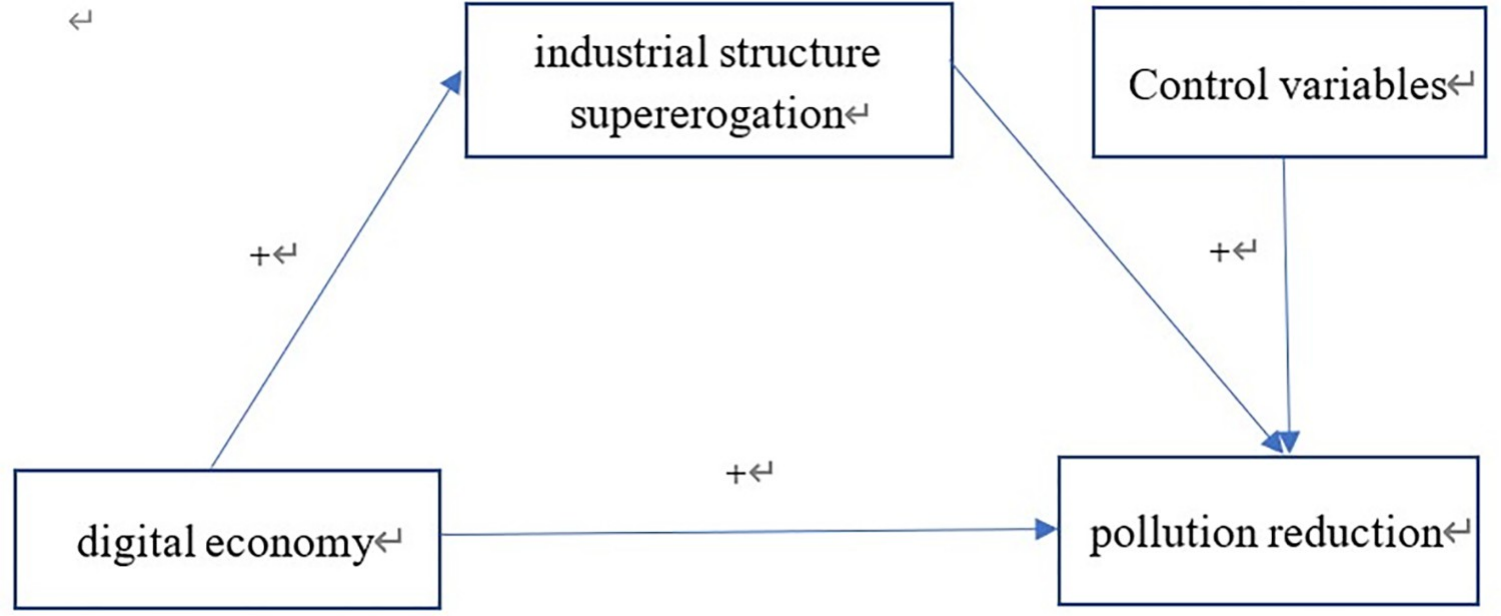
Hypothesized conceptual model and research framework.

Based on the above arguments, we propose the theoretical model shown in Fig 2.

## Empirical analysis

This section first utilizes the coupling coordination model to measure and analyse the connections among the digital economy, industrial structure supererogation and pollution reduction and then further uses the panel data analysis method to analyse these relationships in more detail.

### Sources of data

The data in this article are mainly from the China Statistical Yearbook (2010–2020), China Urban Statistical Yearbook (2010–2020), China Industrial Statistical Yearbook (2010–2020), China Environmental Statistical Yearbook (2010–2020), China Tertiary Industry Yearbook (2010–2020), China Information Industry Yearbook (2010–2020) and EPS database. Some missing data use statistical analysis methods to obtain surrogate values.

### Variable selection and summary statistics of variables

#### Determination of comprehensive evaluation system

A scientific and rational evaluation system is necessary for coupled and coordinated analysis, and the principles of data availability, representativeness, and system relevance must be followed. Based on previous studies, this paper establishes an index system for China’s digital economy-industrial structure supererogation-pollution emission reduction coupling coordination. We divide the digital economy into 5 aspects [36–38]. Regarding the selection of environmental pollution indicators, because different industries have different pollution characteristics, this paper selects from three aspects, and we follow Gan et al. for industrial structure supererogation [39]. Then, we use the entropy weight method to obtain a comprehensive score of each subsystem. Since pollution discharge is a negative indicator, the negative indicator is converted to a positive indicator prior to the entropy analysis. The details of the index system are presented in Table 1.

**Table 1:** Index system of the three subsystems.

Subsystem	Indices	Weight	Attribute
The development of digital economy	total capita telecom business volume	0.1407	+
	total capita postal business volume	0.1577	+
	the number of employees in the information industry	0.2763	+
	the number of internet users at the end of the year	0.3321	+
	the number of mobile phone users at the end of the year	0.0931	+
Industrial structure supererogation	the ratio of the output value of the tertiary industry to the output value of the secondary industry	1	+
Environment pollution	the emissions of industrial sulfur dioxide	0.7168	-
	industrial wastewater	0.2034	-
	industrial soot	0.0798	-

In addition to the abovementioned variables, such as the digital economy, industrial structure supererogation, and pollution emission reduction, this paper selects economic development (pergdp), economic openness (openness), innovation capability (inno), technology employees, government intervention (gs), human resources (hr) and financial resources (fin) that affect pollution emissions. Their statistical characteristics are presented in Table 2.

**Table 2:** Descriptive statistics of the variables.

Variables		Size	Mean	Sd	Min	Max	Explanations
**Explained variable**	**Env**	2780	0.9398	0.0629	0.2791	0.9999	Pollution reduction
**Core explanatory variable**	**Digit**	2780	0.0246	0.0464	0.0006	0.6038	The development level of digital economy
**Mediating variable**	**ISS**	2780	0.8739	0.5636	0.0943	8.0831	Industrial structure supererogation
**Control variables**	**pergdp**	2780	0.3830	0.1674	0.0386	1.9803	per capita gross domestic product
	**open**	2780	1.8308	1.8238	0.0002	19.2392	the ratio of actual utilized FDI to GDP
	**lninno**	2780	8.4053	1.1734	4.6052	13.4765	number of scientific research and technology employees
	**gs**	2780	0.0772	0.0635	0.0042	1	the ratio of government public budget revenue to GDP
	**lnhr**	2780	10.6308	0.7263	7.6497	13.1337	the number of employees in the education industry
	**fin**	2780	1.3152	0.9612	0.0753	15.3561	the ratio of the loan balance of financial institutions to GDP

### Coupling coordination analysis

#### Coupling model

The degree of coupling can be used to measure how much two or more systems influence and affect one another. In this paper, the coupling degree model is used to reflect the internal synergy mechanism among the three subsystems of the digital economy, industrial structure supererogation and pollution reduction in 278 cities. The coupling function is expressed as follows:

$$C_{i}={\left\{\frac{f(x)*g(y)*h(z)}{{\left[\frac{f(x)+g(y)+h(z)}{3}\right]}^{3}}\right\}}^{1/3}$$

where Ci represents the coupling degree of the digital economy, advanced industrial structure and pollution reduction in the ith city, and its range of values is [0, 1]. The closer the value of Ci is to 1, the greater the coupling degree of the three subsystems is, and the more the systems can promote each other; in contrast, a lower coupling degree means that the systems cannot promote each other’s development.

#### Coupling coordination model

The coupling coordination model is necessary to further understand the links among the systems because the coupling degree model cannot accurately depict the degree of coordinated development among subsystems. The coupling coordination degree function is expressed as follows:

$$R_{i}=\sqrt{C_{i}*T_{i}},\;T_{i}=\alpha \;f(x)+\beta \;g(y)+\gamma \;h(z)$$

where Ri stands for the degree of coupling coordination of the digital economy, industrial structure supererogation and pollution reduction in the ith city, the value range is [0, 1], and its value represents the level of coordination among the three systems. Ti represents the comprehensive evaluation index. Finally, α, β, and γ are undetermined coefficients, which represent the contribution of the digital economy, industrial structure supererogation and pollution reduction, respectively, to the overall development level and are set to be equally important in this paper based on earlier research findings [40], so α = β = γ = 1/3. The development stages of the coupling coordination are divided according to the degree of coupling coordination (Table 3).

**Table 3:** Evaluation criteria of the degree of coupling coordination.

Range	Category	Assessment Grade
0–0.29	Extreme incoordination	Non-coordination
0.30–0.39	Moderate incoordination	
0.4–0.49	Mild incoordination	
0.50–0.59	Pre-primary coordination	Transitioning
0.60–0.69	Primary coordination	
0.70–0.79	Moderate coordination	Coordination
0.80–0.89	Good coordination	
0.9–1	Excellent coordination	

#### Analysis of the degree of coupling coordination

**Fig 3:**
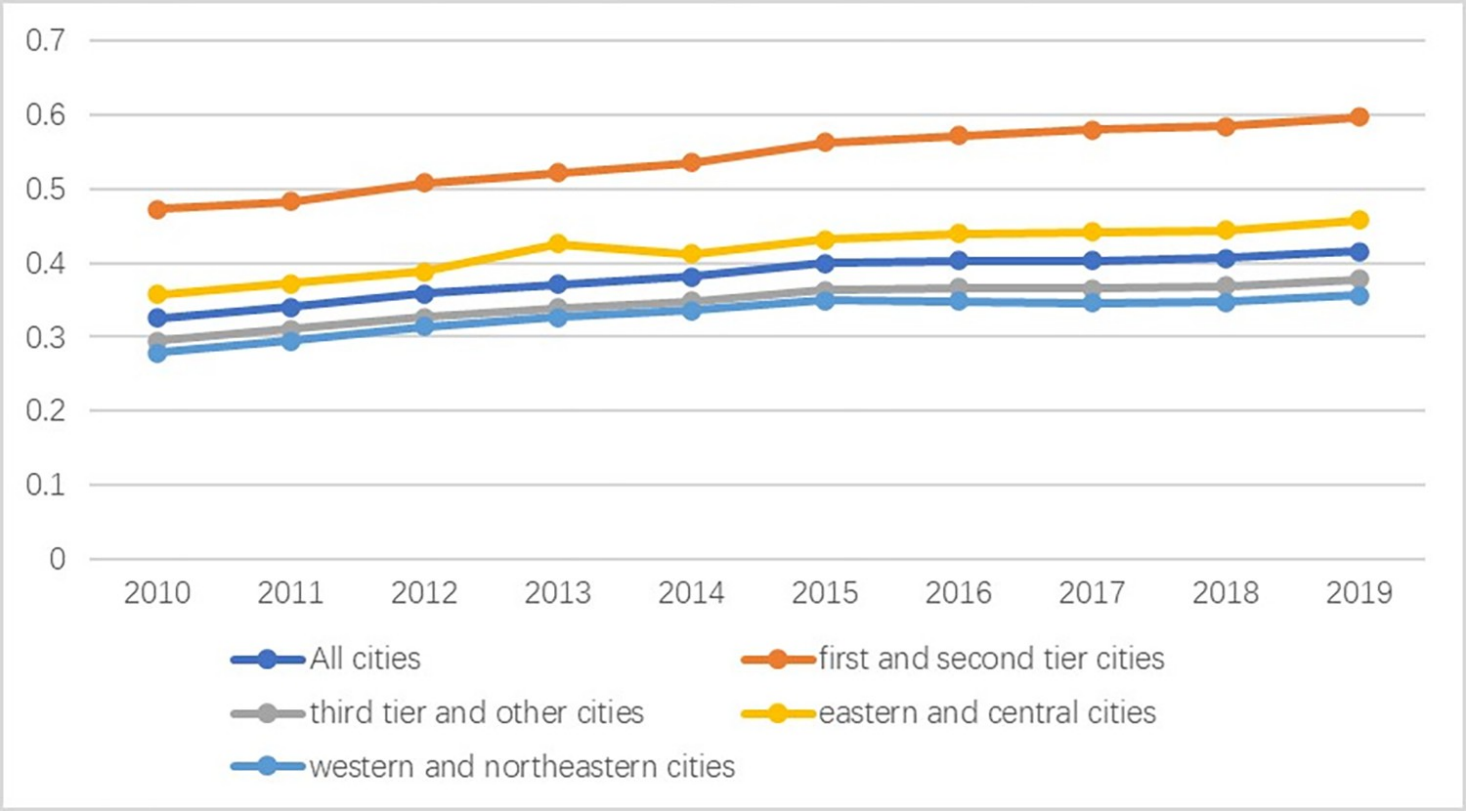
Cities’ degree of coupling of the digital economy, industrial structure supererogation and pollution reduction.

**Fig 4:**
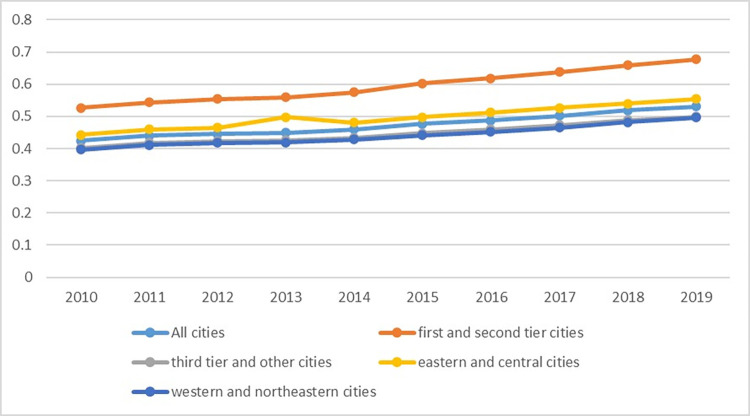
Cities’ degree of coordination of the digital economy, industrial structure supererogation and pollution reduction.

To enhance the stability of the results, this paper carries out dimensionless processing of the indicators. Figs 3 and 4 illustrate the calculation results of the coupling degree and the coupling coordination degree scores of the digital economy, industrial structure supererogation and environmental pollution reduction in 278 cities from 2010 to 2019, which are based on the aforementioned formula and data. To better distinguish the coupling and mutual influence among the growth of the digital economy, advanced industrial structure and pollution reduction, we divide China’s economy into eastern, central, western and northeastern regions according to the classification standards of the National Bureau of Statistics of China and divide Chinese cities into first-tier, second-tier, third-tier and others according to their political influence, economic impact, culture and population. First-tier cities mainly refer to Beijing, Shanghai, Guangzhou, Tianjin and Shenzhen. Second-tier cities generally correspond to provincial capital cities, and third-tier cities refer to other influential cities in each province [38]. Considering factors such as the number of samples, the stage of regional economic development and the characteristics of industrial structure, this paper combines the eastern and central cities into one category and the western and northeastern cities into another category. First-tier and second-tier cities are combined into one category, and third-tier and other cities are combined into another category.

Overall, the average coupling degree of the digital economy, industrial structure supererogation and pollution reduction increased from 0.33 in 2010 to 0.41 in 2019 (Fig 3). This indicates that the interaction is gradually growing, reaching its highest level in 2019 but that there is still much room for improvement. All regions showed a gradual upwards trend, but the eastern and central regions increased from 0.35 in 2010 to 0.46 in 2019, which is much greater than the increase in the western and northeastern regions from 0.28 in 2010 to 0.36 in 2019. This shows obvious regional heterogeneity. In terms of the city development level, the coupling degree among the digital economy, industrial structure supererogation and pollution reduction all showed a gradual upwards trend, but the degree between first- and second-tier cities was significantly greater than that between third-tier and other cities. The coupling degree in first- and second-tier cities increased from 0.47 in 2010 to 0.6 in 2019, showing that the coupling of the three systems has only progressed from preprimary to primary coordination and has huge potential for development. The coupling degree of third-tier and other cities increased from 0.29 in 2010 to 0.38 in 2019. These results indicate obvious city-level heterogeneity in coupling degree due to urban resource endowment, economic foundation, policy measures, and location conditions.

The coupling model only reflects the degree of interaction among different subsystems, whereas the coupling coordination analysis further reflects the coordinated relationship between systems based on the coupling degree. Overall, from 2010 to 2019, coupling coordination among the digital economy, industrial structure supererogation and pollution reduction basically showed a trend of rising fluctuations, and the average value increased from 0.42 in 2010 to 0.53 in 2019 (Fig 4). The coupling coordination increased from a mild lack to preprimary coordination, indicating that the digital economy, industrial structure supererogation and pollution reduction were positively correlated but that overall coordination still needs improvement. The degree of coupling coordination also showed a gradual upwards trend from a regional perspective, which is similar to the characteristics of the coupling degree. The coupling coordination degree of the eastern and central regions increased from 0.44 in 2010 to 0.55 in 2019, which is much higher than the increase from 0.40 in 2010 to 0.50 in 2019 in the western and northeastern regions. These results also show obvious regional heterogeneity. In terms of the city development level, the coupling coordination among the digital economy, industrial structure supererogation and pollution reduction showed a gradual upwards trend in all cities, but the coupling coordination degree of first- and second-tier cities was much higher than that of third-tier and other cities. The coupling coordination degree in first- and second-tier cities increased from 0.53 in 2010 to 0.68 in 2019, indicating that the coordination of the three systems has only completed the transition from preprimary to primary coordination, with huge potential remaining for development. The coupling coordination degree of third-tier and other cities increased from 0.40 in 2010 to 0.50 in 2019, showing obvious heterogeneity at the city level.

### Direct effect analysis

#### Benchmark regression results

To control for the macroeconomic environment and differences between individuals who do not change over time, in accordance with the Hausman test, a double fixed-effects model is employed for regression using the following equation:

$$Env_{it}=\alpha_{0}+\alpha_{1}*Digit_{it}+\alpha_{2}*X_{it}+\mu_{i}+\lambda_{i}+\varepsilon_{it}$$ (1)

where i is the number of the city and t is the year. Envit stands for the pollution reduction in year t of city i, Digit_it_ stands for the development level of the digital economy in year t of city i, X represents the control variable, μ represents the urban effect that is not affected by time, λ represents the time effect that is not affected by the city, and ε represents a random interference term. To reduce heteroscedasticity, we use the natural logarithm of innovation ability and human resources. Table 4 shows the results of the benchmark model. The regression results of Models 1 and 2–7 show that the digital economy’s coefficients are all significantly positive at the 1% level. This preliminarily shows that the digital economy’s development can reduce pollution emissions; that is, Hypothesis H1 holds. Since the environmental reduction index selected in this study is a comprehensive index of three major pollutants and a higher value means less pollution and since the digital economy’s coefficients are significantly positive, this means that the higher the level of digital economy development is, the stronger the inhibitory effect on pollution emissions is. This is mostly because a more developed digital economy leads to a more digitalized and computerized society as a whole. Therefore, the industrial structure becomes more advanced. In addition, enterprise resource utilization becomes more efficient, driving the industry to develop in a green and low-pollution direction, hence reducing pollution emissions.

**Table 4:** Results of the benchmark model.

Variable	Model 1	Model 2	Model 3	Model 4	Model 5	Model 6	Model 7
Digit	0.29646*** (7.99)	0.29641*** (7.91)	0.30312*** (7.95)	0.30633*** (8.03)	0.30629*** (8.03)	0.30701*** (8.04)	0.30751*** (8.04)
Lninno		0.00002	0.00069	0.00035	0.00038	0.00064	0.00063
Lnhr			-0.00756	-0.00732	-0.00704	-0.00672	-0.00657
Fin				-0.00524	-0.00485	-0.00457	-0.00448
Gs					-0.06126	-0.06116* (-1.75)	-0.06281* (-1.79)
Pergdp						0.00302	0.00283
Open							0.00024
Constant	0.92203*** (476.59)	0.92205*** (270.69)	0.93295*** (77.93)	0.93576*** (77.46)	0.93897*** (76.89)	0.93778*** (74.24)	0.93714*** (73.55)
Year effect	Yes	Yes	Yes	Yes	Yes	Yes	Yes
City effect	Yes	Yes	Yes	Yes	Yes	Yes	Yes
N	2780	2780	2780	2780	2780	2780	2780

Notes: The t-statistic is in parenthesis

***, *, and *indicate statistically significant at 1%, 5%, and 10%, respectively.

For the control variables, only the coefficient of government intervention is statistically negative and significant, indicating that the increase in the proportion of government public budget revenue to GDP will significantly increase pollution emissions, mainly because increased government budget revenue will squeeze the income of enterprises and residents, which will lead to insufficient enterprise investment in green technologies. It will also delay residents’ replacement of high-energy-consuming products and ultimately lead to an increase in pollution emissions.

### Robustness of the benchmark model

This study tests the robustness of the benchmark model from four different angles: by adding a control variable, replacing the core explanatory variable, changing the estimation method and considering endogeneity. Table 5 presents the results. First, to avoid the endogeneity problem caused by two-way causality and some unobservable factors, according to the previous studies by Huang Qunhui et al. [41] and Zhao et al. [42], this paper uses historical data on post and telecommunications in each city in 1984 as the instrumental variable of the comprehensive index of digital economic development. On the one hand, as the traditional communication technology, the local historical telecommunications infrastructure affects the subsequent application of internet technology in terms of the technical level and usage habits. On the other hand, the impact of traditional telecommunication tools, such as fixed telephones, on economic development gradually diminishes as the frequency of use decreases, satisfying exclusivity. The original data of the selected instrumental variable are in the form of a cross section and cannot be directly used for the metrological analysis of panel data. Referring to Nunn and Qian’s (2014) treatment of this problem [43], a variable that changes with time is introduced to construct the panel instrumental variable. Specifically, the number of national internet users in the previous year per 10000 people in each city in 1984 is used to construct interactive terms, which are used as the instrumental variables of the urban digital economy index in that year for each city for the endogeneity test [41, 42]. From the results of 2SLS in Table 5, we can see that after endogeneity is considered, the effect of the digital economy on pollution reduction is still significant at the 5% level. In addition, for the test of the original hypothesis "insufficient identification of instrumental variables", the Kleibergen-PaaP rk LM statistic P value is 0.000, which significantly rejects the original hypothesis. In the test of weak recognition of instrumental variables, the Wald F statistic of the Kleibergen-PaaP rk exceeds the critical value of the stock Yogo weak recognition test at the level of 15%. The above test shows that it is generally reasonable to select the cross term between the number of telephones in each city from historical data and the number of national internet users in the previous year as the digital economy index instrumental variable. We also take Germann and Grewal’s advice to mitigate endogeneity concerns by including other control variables in our model [44]. As an additional test of robustness, we incorporate extra export control variables into the model, which also confirms the proposed hypothesis. Third, since the total amount of post and telecommunications activity can somewhat reflect the development level of the digital economy [38], this paper re-estimates the benchmark model by taking the total amount of such activity as a surrogate variable for the core explanatory variable. Finally, this study employs a dynamic panel model to test the robustness of the direct effect.

**Table 5:** Results of the robustness test of the benchmark model.

Variable	Adding control variable		Replacing core variable	2-stage GMM	2SLS
Digit (-1)				-0.39540*** (-6.98)	
Digit	0.30710*** (8.03)			1.19717*** (3.15)	0.98720**
Lnpost			0.00298* (1.78)		
Controlled	YES		YES	YES	YES
Constant	0.94151*** (51.01)		0.90022*** (44.07)		
City effect	YES		YES	YES	
Year effect	YES		YES	YES	
P (Sargan)				0.131	
P (Hansen)				0.183	
Cragg-Donald Wald F statistic					22.04(8.96)
Kleibergen‒Paap Wald rk F statistic					12.34(8.96)
Kleibergen‒Paap rk LM statistic					12.10***
The results of first stage of 2SLS					
IV					-0.00385***
Controlled					YES
F					12.34***
N		2780	2780	2224	2780

Notes: The t-statistic is in parentheses

***, *, and * indicate statistical significance at the 1%, 5%, and 10% levels, respectively.

Table 5 shows that after endogeneity concerns are considered, the coefficient of the digital economy is still significant at the 5% level, which is basically consistent with the benchmark regression results. When additional control variables are added, the digital economy’s coefficient is still significantly positive, confirming the robustness of the benchmark regression conclusion. When reregressed with the surrogate variables of the core explanatory variable, the coefficient of the surrogate variable is significantly positive. In addition, from the estimation results of the 2-step GMM, the P values of the Sargan and Hansen tests are both above 0.1, indicating that the instrumental variables are effective, and the regression results of the GMM once again confirm the robustness of the benchmark model.

#### Heterogeneity analysis

The results of the benchmark model indicate that the development of the digital economy is generally good for pollution emission reduction. Is this effect universal in different regions and at different city levels? To this end, we conduct the following subregional and subcity level regression analysis. Table 6 demonstrates that the digital economy has a significant effect on pollution reduction in all regions. However, the effect on the western and northeastern regions is greater than that on the eastern and central regions. This is mainly because, in contrast to the former regions, the latter regions’ economies are mostly based on heavy and resource-intensive industry, with high dependence on traditional resources and low energy utilization. In addition, digitalization there is not as high as in the eastern and central regions. In the early stage of the development of the digital economy, digital technology can play a great role in reducing energy use intensity and improving the allocation efficiency of energy resources, thereby significantly reducing pollution emissions [45, 46]. From the perspective of the city development level, the digital economy not only has significant pollution reduction effects on first- and second-tier cities but also on third-tier and other cities. Moreover, the pollution reduction effect on third-tier and other cities is greater than that on first- and second-tier cities. This is mainly due to the large proportion of the service industry in the more developed cities, while the industrial structure of less developed cities is dominated by manufacturing, which is a major source of pollution. Hence, the effect of the digital economy on pollution reduction in the manufacturing industry is greater than that in the service industry.

**Table 6:** Results of the heterogeneity test.

Variable	Eastern & central cities	Western & northeastern cities	First & tier cities	Third tier & other cities
Digit	0.24326***(6.07)	0.68851***(6.62)	0.22678***(3.41)	0.31850***(2.69)
Controlled	YES	YES	YES	YES
Constant	0.93921***(53.59)	0.92785***(47.83)	0.88749***(14.52)	0.95125***(78.55)
City effect	YES	YES	YES	YES
Year effect	YES	YES	YES	YES
N	1650	1130	490	2290

Notes: The t-statistic is in parenthesis

***, *, and *indicate statistically significant at 1%, 5%, and 10%, respectively.

### Indirect effect analysis–Mediation test

To check whether there is a mediation effect of industrial structure supererogation between the digital economy and pollution reduction, we use Eq (1) as a base and industrial structure supererogation as a mediating variable to construct the following model:

$$ISS_{it}=\beta_{0}+\beta_{1}*Dig_{it}+\beta_{2}X_{it}+\mu_{i}+\lambda_{i}+\varepsilon_{it}$$ (2)

$$Env_{it}=\gamma_{0}+\gamma_{1}*Digit_{it}+\gamma_{2}*ISS_{it}+\gamma_{3}X_{it}+\mu_{i}+\lambda_{i}+\varepsilon_{it}$$ (3)

Table 7 shows the mediating effect of industrial structure supererogation between the digital economy and pollution reduction and its robustness test results. Among them, Model 1 verifies that the digital economy is positively related to the intermediary variable industrial structure supererogation. The results of Model 2 indicate that after adding the mediating variable, the regression estimation coefficient of the digital economy drops from 0.30751 to 0.29749, indicating that industrial structure supererogation partially mediates the effect of the digital economy on pollution reduction. The indirect effect through industrial structure supererogation is *β*_1_*γ2, which is 0.01003_._ When an additional control variable is added, the digital economy’s coefficient is significantly positive, which indicates that the benchmark mediation model’s conclusions are solid. When reregressed with the surrogate variables of the core explanatory variable, the surrogate variable’s coefficient is still significantly positive. Again, this confirms that the mediation effect is robust. Since the existence of the mediation effect cannot be determined simply by the significance of the coefficient, we also conduct the Sobel test. The significant test results in Table 8 show that the mediation effect exists.

**Table 7:** Results of the mediation and robustness tests.

Variable	(1) ISS	(2) Env	(3) Replacing explanatory variable	(4) Adding control variable
Digit	1.13082*** (4.5)	0.29749*** (7.76)		0.29750*** (7.76)
ISS		0.00887*** (2.91)	0.01105*** (3.6)	0.00887** (2.89)
Lnpost			0.00304* (1.82)	
Controlled	YES	YES	YES	YES
Constant	0.62544*** (7.47)	0.93159*** (72.41)	0.89310*** (43.62)	0.93145*** (49.66)
City effect	YES	YES	YES	YES
Year effect	YES	YES	YES	YES
Sobel test		5.704***	3.874***	4.877***
N	2780	2780	2780	2780

Notes: The t-statistic is in parentheses

***, *, and * indicate statistical significance at the 1%, 5%, and 10% levels, respectively.

**Table 8:** Results of the heterogeneity of the mediating effect.

Variable	Eastern and central cities		Western and northeastern cities		First- and second-tier cities		Third-tier and other cities	
	ISS	Env	ISS	Env	ISS	Env	ISS	Env
Digit	1.30187*** (8.41)	0.19297*** (4.76)	0.42827 (0.48)	0.68677*** (6.6)	1.31389*** (8.09)	0.14049* (1.99)	0.43578 (0.48)	0.31593** (2.67)
ISS		0.03862*** (5.78)		0.00407 (1.1)		0.06567*** (3.35)		0.00590* (2.07)
Controlled	YES	YES	YES	YES	YES	YES	YES	YES
Constant	0.52745*** (7.79)	0.91884*** (51.94)	0.81800*** (4.93)	0.92453*** (47.09)		0.85033*** (13.85)	0.70977*** (7.56)	0.94706*** (77.19)
City effect	YES	YES	YES	YES	YES	YES	YES	YES
Year effect	YES	YES	YES	YES	YES	YES	YES	YES
Sobel test		8.132***		1.670*		5.463***		3.548***
N	1650	1650	1130	1130	490	490	2290	2290

Notes: The t-statistic is in parentheses

***, *, and * indicate statistical significance at the 1%, 5%, and 10% levels, respectively.

#### Heterogeneity of mediation test

The mediation model shows that digital economy development can reduce pollution emissions through industrial structure supererogation. Is this mediation effect universal in different regions and at different city development levels? To this end, we conduct the following subregional and subcity level regression analyses. Table 8 shows that for the eastern and central regions, after adding industrial structure supererogation, the digital economy’s coefficient drops from 0.24326 to 0.19297, indicating that industrial structure supererogation plays a significant mediating role in the eastern and central regions. The results of the Sobel test also indicate that the mediation effect of industrial structure supererogation is significant at the 1% level. However, for the western and northeastern regions, the regression results show that the mediating effect is not significant, which means that industrial structure supererogation does not play a mediating role in these regions at the significance level of 5%. This is mainly because most of the sectors related to the digital economy are intellectual and capital intensive, and their growth requires a large amount of support from high-quality talent. In contrast to the eastern and central regions, the lack of capacity to develop emerging industries has led to weak support for the growth of the digital economy on industrial structure supererogation. This is similar to our earlier conclusion with the coupling coordination model, which is that coupling coordination among digital economy development, industrial structure supererogation and pollution reduction is low and noncoordinated. From the perspective of city heterogeneity, for first- and second-tier cities, the digital economy’s coefficient dropped from 0.22678 to 0.14049 after the intermediary variable of industrial structure supererogation is added, indicating that industrial structure supererogation plays a significant intermediary role between digital economy development and pollution reduction in first- and second-tier cities but not in the third-tier and other cities. This is consistent with our earlier conclusion using the coupling coordination model. Compared with first- and second-tier cities, third-tier and other cities have less coupling coordination of digital economic development, industrial structure supererogation and pollution reduction and are in the noncoordination stage.

## Discussion

The results of the benchmark regression model show that digital economy development can significantly reduce pollution emissions, which is basically consistent with previous studies [18, 19, 26, 27], mainly because digital economy development improves enterprises’ production efficiency, reduces energy consumption, promotes the growth and formation of emerging industries with low pollution and low energy consumption, and promotes industrial structure supererogation, thereby reducing the intensity of pollution emissions.

From the heterogeneity analysis of regions and cities, the digital economy’s development has a much larger emission reduction effect on regions and cities with abundant energy resources, high pollution and high-energy-consumption industries; that is, its pollution reduction effect has greater marginal utility in the western and northeastern regions, third-tier cities and other cities, mainly because the application of digital technology can greatly improve these regions’ energy efficiency and reduce the energy use intensity of industrial enterprises. This result is consistent with Xie [46] and Guo et al. [25] but not with the findings of Deng and Zhang [3]. From the perspective of the mediating effect and its heterogeneity, industrial structure supererogation can partly mediate the growth of the digital economy and pollution reduction as a whole [47]. However, for the western and northeastern regions, as well as the third-tier and other cities, the mediating effect is not significant. This suggests that the main reason for the digital economy’s high marginal utility in the western and northeastern regions and the third-tier and other cities is not that it has led to the industrial structure supererogation of these regions and cities. Rather, the impact may be caused mostly by increased efficiency in resource use and reduced energy use intensity by industrial enterprises. However, in the eastern and central regions and first- and second-tier cities, digital economy development has indeed significantly accelerated industrial structure supererogation, which has reduced pollution. The main reason for this difference is that emerging industries centred on information technology are generally capital- and intelligence-intensive industries, and their development and growth cannot be achieved without the support of high-quality human capital. Western and northeastern regions and third-tier cities are relatively deficient in production factors such as highly qualified talent, which slows the growth of new industries and industrial structure supererogation.

The results of the econometric model also show that industrial structure supererogation does not play an intermediary role between digital economy development and pollution reduction in the western and northeastern regions and third-tier and other cities. That is, digital economy development, industrial structure supererogation, and pollution reduction are not coordinated. From the results of coupling coordination analysis and the panel data analysis, compared with the eastern and central regions and first- and second-tier cities, the western and northeastern regions and third-tier and other cities lag behind with regard to coupling and coupling coordination degree, which basically remain noncoordinated. The conclusions drawn from the two methods can be mutually confirmed.

## Conclusion

Using panel data of 278 Chinese cities from 2010 to 2019, this article starts by constructing an index of digital economy development and pollution reduction using coupling coordination analysis, fixed effect analysis, and mediating effect analysis to study the direct and indirect effects of the digital economy on pollution reduction and their mechanism from multiple angles. The main findings are as follows. First, the coupling coordination model indicates that the coupling coordination of China’s digital economy, industrial structure supererogation and pollution reduction is still at the primary level, and there is considerable room for improvement. With regard to regional heterogeneity, the coupling coordination of the eastern and central regions is better than that of the rest of China. In terms of city heterogeneity, the coupling coordination degree of first- and second-tier cities is better than that of third-tier and other cities. Second, from the perspective of the direct effect analysis, the digital economy’s development has a positive influence on pollution reduction as a whole and has emerged as a key driver of China’s green development. In terms of regional heterogeneity, the digital economy’s emission reduction effect in the western and northeastern regions is significantly better than that in the eastern and central regions. In terms of cities, the emission reduction effect in third-tier and other cities is significantly better than that in first- and second-tier cities. Third, from the perspective of mediation effect analysis, industrial structure supererogation as a whole can mediate the digital economy’s development and pollution reduction. However, this role is only applicable in the eastern and central regions and is not significant in the western and northeastern regions. In terms of city heterogeneity, industrial structure supererogation can play an intermediary role between digital economic development and pollution reduction in first- and second-tier cities. The mediating effect is not significant in the third-tier and other cities.

### Policy implications

We make the following policy recommendations based on the findings of this study. First, we suggest accelerating digital economy development and improving digital economy infrastructure to provide new momentum to reduce urban pollution. Expediting the construction of new infrastructure, such as 5G, cloud computing, blockchain, and data centres, will encourage the deep integration of the digital economy with cities, industries, and enterprises and upgrade the industrial structure. We also recommend broadening the application of the digital economy and digital technology in various fields of manufacturing, accelerating enterprises’ digital transformation, and improving energy utilization efficiency and enterprise production efficiency to reduce pollution emissions.

Second, the central government should implement a regional differentiation strategy and allocate more digital resources to the western and northeastern areas to help the local government implement a differentiated digital economy development strategy. It should formulate preferential fiscal and tax policies to promote digital economy development in the western and northeastern regions, strengthen financial investments in science and technology in third-tier and other cities, vigorously cultivate digital economic technical talent and application-oriented innovative talent, and encourage the flow of digital economic talent and capital resources to the west and northeast regions to maximize the digital economy’s marginal utility in promoting pollution reduction. Each region should clarify its digital economy development strategy based on local advantages and invest more resources in industrial digitalization. Around the core industries of the digital economy, the local government must encourage and help leading enterprises build industrial chains and industrial internet platforms along the digital economy industrial chain; promote key digital economy park construction; and accelerate cloud computing, big data, artificial intelligence and other industrial digital resource agglomeration to promote industrial structure supererogation.

Finally, we recommend coordinating the development of the regional digital economy. We should strengthen regional digital economy industry connections, regional data centres and platform construction and make full use of digital technology in promoting industrial structure supererogation. We should also raise the level of digital technology application in the eastern and central regions, combine the low-cost and resource advantages of the western and northeastern regions with the technological, market and digital industry advantages of the eastern and central regions to form a mechanism for complementary advantages and coordinated development.

### Limitations and future research directions

This study has limitations. First, it uses panel data to analyse temporal characteristics and city differences but does not consider the spatial correlation and temporal heterogeneity among different cities. In the future, dynamic spatial panel data can be used for analysis to increase spatial correlation and temporal heterogeneity. Second, due to limitations on data availability, the selection of indicators in this paper is mainly based on macro variables; as a result, the mechanism of digital economy development that affects pollution reduction cannot be fully ascertained. For example, production efficiency improvement is also a main path for the digital economy to affect pollution reduction, which can be studied at the micro level in the future. Finally, the digital economy development indicators in this paper are not comprehensive due to limited data availability. In the future, with more available data, we should continue to improve the measurement method of digital economy development.

## Supporting information

S1 Data (ZIP)
